# Determination of Regorafenib monohydrate (colorectal anticancer drug) solubility in supercritical CO_2_: Experimental and thermodynamic modeling

**DOI:** 10.1016/j.heliyon.2024.e29049

**Published:** 2024-04-15

**Authors:** Gholamhossein Sodeifian, Ratna Surya Alwi, Fatemeh Sodeifian, Solmaz Amraee, Mohammadreza Rashidi-Nooshabadi, Fariba Razmimanesh

**Affiliations:** a Department of Chemical Engineering, Faculty of Engineering, University of Kashan, 87317-53153, Kashan, Iran; b Laboratory of Supercritical Fluids and Nanotechnology, University of Kashan, 87317-53153, Kashan, Iran; c Modeling and Simulation Centre, Faculty of Engineering, University of Kashan, 87317-53153, Kashan, Iran; d Research Centre for Computing, National Research and Innovation Agency (BRIN), Jl, Raya Jakarta-Bogor KM 46 Cibinong, Indonesia; e School of Medicine, Shahid Beheshti of Medical Sciences, Iran; f Department of Pharmacology, School of Medicine, Kashan University of Medical Sciences, 87159-88141, Kashan, Iran

**Keywords:** Regorafenib monohydrate, Supercritical carbon dioxide, Solubility, Anticancer, Thermodynamic modeling

## Abstract

In this study, the solubilities of *Regorafenib monohydrate* (*REG*), a widely used as a colorectal anticancer drug, in supercritical carbon dioxide (ScCO_2_) were measured under various pressures and temperature conditions, for the first time. The minimum value of *REG* in mole fraction was determined to be 3.06×10^−7^, while the maximum value was found to be 6.44×10^−6^ at 338 K and 27 MPa. The experimental data for *REG* were correlated through the utilization of two types of models: (1) a set of 25 existing empirical and semi-empirical models that incorporated 3–8 parameters according to functional dependencies, (2) a model that relied on solid-liquid equilibrium (*SLE*) and the newly improved association models. All of the evaluated models were capable of generating suitable fits to the solubility data of *REG*, however, the average absolute relative deviation (AARD) of Gordillo et al. model (AARD=13.2%) and Reddy et al. model (AARD=13.5%) indicated their superiority based on AARD%. Furthermore, solvation and sublimation enthalpies of *REG* drug were estimated for the first time.

## Highlights

• The solubility of *Regorafenib monohydrate* (*REG*) in ScCO_2_ was measured for the first time.
• Mole fractions ranged from 3.06-×-10^−7^ to 6.44-×-10^−6^.
• The solubility data were correlated by 25 density-based models, and *SLE*.
• The method of evaluating experimental data self-consistency was assessed.
• The total (Δ*H*_tot_), vaporization (Δ*H*_vap_), & solvation (Δ*H*_sol_) enthalpies of *REG* - were determined.

## 1 Introduction

*Regorafenib*, also known as *REG [4-(4-(3-(4-chloro-3-(trifluoromethyl) phenyl) ureido)-3-fluorophenoxy)-N-methylpicolinamide]*, is an orally administered multikinase inhibitor that was synthesized by the Bayer business. It received approval from the U.S. Food and Drug Administration (FDA) in 2012 under the trade name Stivarga® [[1], [2], [3]]. The utilization of *REG* has been demonstrated in the management of gastrointestinal stromal tumors and metastatic colorectal cancer. Additionally, it has been suggested as a second-line therapeutic option for the management of advanced hepatocellular carcinomas that exhibit resistance to or intolerance to sorafenib. *REG* is found in multiple crystal forms, with the monohydrate form (REG·H_2_O) being chosen for commercial purposes [1].

The effectiveness of REG is significantly constrained by its low oral bioavailability, which is attributed to its inadequate solubility in water, as indicated by a solubility value of 0.12 μg/mL at pH 6.5 and 22–23 °C [4,5]. To attain an adequate drug concentration, the administration of REG is conducted orally at a dosage of 160 mg per day within a clinical setting. However, this practice exacerbates the occurrence of adverse effects and introduces a certain level of safety concerns for the patients [1].

In the last few years, there has been a notable surge in research endeavors focused on enhancing targeted drug delivery systems, motivated by the escalating demand within the pharmaceutical sector. The acknowledgment of the importance of parameters such as solubility and bioavailability is highly pertinent, emphasing the need to advance particle engineering methods in order to attain optimal regulation of particle size [[6], [7], [8], [9], [10], [11], [12], [13], [14], [15], [16], [17], [18], [19], [20]]. A substance that displays characteristics that lie between those of a liquid and a gas is categorized as a supercritical fluid (SCF) if it exceeds its critical pressure

and temperature. A lot of interest has been shown in using supercritical fluids (SCFs) in a variety of industrial processes, such as nanoparticle formation [[20], [21], [22], [23]], essential oil [[24], [25], [26], [27], [28], [29]], seed oil [[30], [31], [32], [33]], solubility [34], impregnation [35,36], optimization and mathematical modeling [26,37] and polymer synthesis [38].

The current study gives useful information on how to choose the best method for making drug nanoparticles or micro particles using supercritical technology, which eventually leads to a lower dose of medication. In order to assist further investigation, it is crucial to acquire experimental solubility data for the pharmaceutical compound. We have reported experimental solubility in ScCO_2_ of various pharmaceutical compound, namely, Buprenorphine hydrochloride [39], Nilotinib hydrochloride monohydrate [40], Fexofenadine hydrochloride [41], Hydroxychloroquine sulfate [42], Ibrutinib [43], Riluzole [44], Palbociclib [45],Rivaroxaban [46], Crizotinib [47], Prazosin hydrochloride [48], Pazopanib hydrochloride [49], Metoclopramide hydrochloride [50], empagliflozin [51]Pantoprazole sodium sesquihydrate [52], teriflunomide [53], Pholcodine [54], Dasatinib monohydrate [55], Clemastine fumarate [56], Quetiapine hemifumarate [57], Losartan potassium, Cozaar [58], Galantamine [59], Ketoconazole [36], Amlodipine Besylate [60], Minoxidil [9], Tamsulosin [13], Triamterene (2,4,7-Triamino-6-phenylpteridine) [7], Sodium Valproate [15], Lansoprazole [10], Azathioprine [6], Sorafenib tosylate [61], Sunitinib malate [20], Esomeprazole [62], Repaglinide [63], Oxcarbazepine [64], Sertraline hydrochloride [14], Imatinib mesylate [8], Loratadine [65], Letrozole [66], Ketotifen fumarate [12], Amiodarone hydrochloride [67], Aprepitant [68], Triamcinolone acetonide [69], and Codeine phosphate [70]. However, the literature does not provide information regarding the solubility of *Regorafenib monohydrate* (*REG*) in supercritical carbon dioxide (ScCO_2_). Therefore, this study aims to measure and model the solubility of *REG* in ScCO_2_, for the first time.

The obtained solubility data of *REG* were subsequently analyzed by employing two distinct models, namely 25 existing density-based models. These models were further categorized into three categories based on their function dependencies. Group I, consisting of Mendez-Teja [71], Bartle et al. [72], Jafari et al. [73], Hozhabr et al. [74], Keshmiri et al. [75], Khansary et al. [76], Jouyban et al. [77], and Sodeifian et al. [17], for temperature (T), CO_2_ density (*ρ*_1_), and pressure (P) as independent variables in their models. The study utilized models from Group II, which consisted of Kumar-Johnston [78], Alwi-Garlapati [79], Chrastil [80], Andonova-Garlapati [81], Sung-Shim [82], Garlapati-Madras [83], Del Valle-Aguilera [84], Bian et al. [85], Adachi-Lu [86], Sparks et al. [87], Si-Moussa et al. [88], and Belghait et al. [89]. These models incorporated inde-pendent variables such as CO_2_ density (*ρ*_1_) and temperature (*T*). Group III, namely Reddy et al. [90], Mitra-Wilson [91], Reddy-Garlapati [92], Reddy-Garlapati [92], Gordillo et al. [93], and Yu et al. [94], as employed temperature (*T*) and pressure (*P*) as independent variables. Furthermore, models with cat-egories based on solid-liquid equilibrium (*SLE*) like association theory models developed by Rajasekhar-Madras [95], and new modified association models. Finally, the total (Δ*H*_tot_), vaporization (Δ*H*_vap_), and solvation (Δ*H*_sol_) enthalpies for the *REG*-ScCO_2_ binary system were estimated as part of this work.

## 2 Experimental

### 2.1 Materials

*Regorafenib monohydrate* (*REG*), purchased from Temad CO. (Karaj, Iran), was obtained as a crystalline white powder with a purity level of 99.9-%. Table 1 provides additional details regarding the physicochemical parameters of Regorafenib monohydrate. The storage cylinder contained compressed carbon dioxide (CO_2_) with a purity level of 99.99-%, which was provided by Fadak Company located in Kashan, Iran. The collecting solvent for *REG* after the solubility process was chosen to be *Dimethyl sulfoxide* (DMSO), an analytical reagent grade with a minimum purity of 99.5-%. This particular DMSO was manufactured by Merck (Darmstadt, Germany).

**Table 1:** The molecular structure and physicochemical properties of the materials examined in this study.

Compound	Structure	*M* (g/mol)	λ_max_ (nm)	CAS number	Minimum purity
*Regorafenib monohydrate* (*REG*) (C_21_H_17_ClF_4_N_4_O_4_)		500.83	275	1019206-88-2	99.50%
Carbon dioxide (CO_2_)		44.01		124-38-9	99.99%
*Dimethyl sulfoxide* DMSO (C_2_H_6_OS)		78.13		67-68-5	99.5%

**Table 2:** Crystalline *Regorafenib monohydrate* (*REG*) solubility in ScCO_2_ at different pressures and temperatures. The experimental standard deviation was obtained by $\left(y_{k}\right)=\sqrt{\frac{\sum_{j=1}^{n}{\left(y_{j}-\bar{y}\right)}^{2}}{n-1}}$. Expanded uncertainty (U) and the relative combined standard uncertainty *combined/y* are defined, respectively, as follows; *U* = *k* ∗ *u*_*combined*_(*k* = 2), and $u_{combined}/y=\sqrt{\sum_{i=1}^{N}{\left(P_{i}u\left(x_{i}\right)/x_{i}\right)}^{2}}$. In this research, *u* (*x*_*i*_) was considered as the standard uncertainties of temperature, pressure, mole fraction, volumes and absorption. $P_{i}$, sensitivity coefficients, are equal to the partial derivatives of y equation with respect to the *x*_*i*_.

Temperature (*K*)^*a*^	Pressure (*bar*)^a^	Density of CO_2_ (*kg/m*^3^) [96]	*y*_2_ × 10^5^ (mole fraction)	Experimental standard deviation *S* × (10^6^)	S (equilibrium solubility) (*g/L*) × 10	Expanded uncertainty of mole fraction (10^5^*U*)
308	120	769	0.125	0.05	0.106	0.058
	150	817	0.173	0.07	0.155	0.078
	180	849	0.192	0.02	0.180	0.086
	210	875	0.235	0.08	0.226	0.104
	240	896	0.297	0.11	0.292	0.131
	270	914	0.313	0.10	0.315	0.138
318	120	661	0.101	0.03	0.072	0.046
	150	744	0.121	0.04	0.098	0.054
	180	791	0.143	0.02	0.124	0.064
	210	824	0.317	0.05	0.287	0.141
	240	851	0.342	0.02	0.319	0.151
	270	872	0.389	0.03	0.372	0.171
328	120	509	0.064	0.02	0.036	0.030
	150	656	0.098	0.04	0.070	0.044
	180	725	0.126	0.06	0.100	0.056
	210	769	0.392	0.03	0.331	0.174
	240	802	0.421	0.07	0.371	0.186
	270	829	0.431	0.06	0.392	0.191
338	120	388	0.031	0.01	0.013	0.014
	150	557	0.062	0.03	0.037	0.028
	180	652	0.102	0.01	0.072	0.044
	210	710	0.443	0.12	0.346	0.197
	240	751	0.497	0.09	0.410	0.220
	270	783	0.644	0.06	0.553	0.284

The coverage factor, k = 2 corresponds to a confidence level of approximately 95 %.

a Standard uncertainty *u* is herein set to *u*(*T*) = ±0.1 K and *u*(*p*) = ±1 bar.

### 2.2 Measurements by supercritical carbon dioxide

**Fig. 1:**
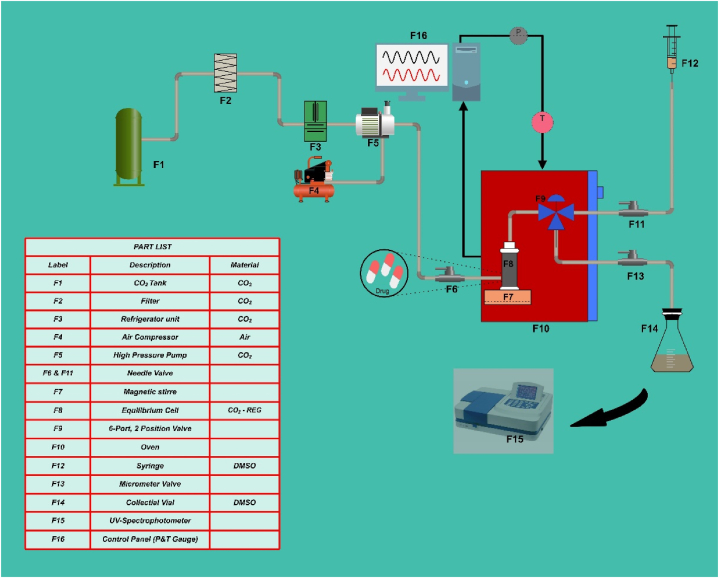
The experimental configuration employed in this study.

The solubility of *REG* in ScCO_2_ was measured using a pilot-scale apparatus, as depicted in Fig. 1. The equipment was constructed using 316 stainless steel and had a thickness of 1/8 inch. An F-1 CO_2_ cylinder, an F-2 needle valve, an F-3 molecular sieve filter, an F-4 refrigeration unit, an F-5 high-pressure pump, an F-6 air compressor, an F-7 oven, an F-8 magnetic stirrer, an F-9 high-pressure equilibrium cell, an F-10 3-position valve, and an F-16 back-pressure valve were all part of the apparatus.

The experiments were conducted within a pressure range of 12–27 MPa and at temperatures spanning from 308 K to 338 K. The methodology employed to determine the solubility of *REG* in ScCO_2_ has been comprehensively elucidated in a prior investigation [[6], [7], [8],^17^,^48^,^49^,^54^,^97^,^98^]. In each experimental trial, a consistent amount of 1 g of the *REG* drug was administered into the immobile cell, which was subsequently positioned inside a thermal chamber. The aforementioned measure was enacted with the purpose of ensuring that the temperature remains within a narrow range of ±0.1 K from the designated experimental point. At both cell ends, stainless-steel sintered filters were added to limit undissolved *REG*. After that, high-pressure CO_2_ was poured into the cell and allowed to sit for 70 min in order to obtain saturation. Based on earlier studies, we know that 60 min is sufficient to achieve balance. The isobaric-isothermal category includes this kind of measurement [99]. Throughout the experiment, the target temperatures and pressure levels were kept within ±0.1 K and ±0.1 MPa, respectively, for every data point. Following the triplicate execution of the experiments, the average solubility values were computed.

After reaching equilibrium, we used a 2-position 6-way port valve to collect 600 μL of saturated ScCO_2_ samples. The materials were thereafter transferred into a vial that was pre-filled with dimethyl sulfoxide (DMSO). The flow rate was managed by means of a micrometer valve. Each experiment was finished with the flow line being purified with 1 mL of DMSO after 5 mL of the solution had been collected. Using a UNICO-4802 UV–Vis spectrophotometer set to a peak wavelength (*λ*_max_) of 275 nm, we examined the absorbance of *REG* in DMSO for solubility assessment. The solubility of *REG* in ScCO_2_ was determined according to a given equation. The amount of *REG* dissolved in ScCO_2_, denoted as (*y*_2_), was measured in terms of mole fraction. The solubility was determined by employing a calibration curve, which exhibited a high regression coefficient of 0.998. The quantification of the dissolved substance was accomplished through the measurement of UV absorbance. The calculation of the moles of *REG* (*n*_*REG*_) and CO_2_ (*n*_1_) in the loop was performed as follows:

(1) $$n_{REG}=\frac{C_{REG}\left(\frac{g}{L}\right).V_{REG}\left(L\right)}{M_{REG}\left(\frac{g}{mol}\right)}$$

(2) $$n_{1}=\frac{V_{1}\left(L\right).\rho_{1}\left(\frac{g}{L}\right)}{M_{1}\left(\frac{g}{mol}\right)}$$

The concentration of *REG* (g/L) in the collection vial is denoted as *C*_*REG*_. The sampling loop and the collection vial are represented by *V*_1_ (*L*) and *V*_*REG*_ (*L*), respectively. The symbols *M*_1_ and *M*_*REG*_ represent the molar masses of carbon dioxide (CO_2_) and a substance denoted as *REG*, respectively. The method out-lined in references [7,17] was employed to determine the equilibrium mole fraction of *REG*, denoted as *y*_2_, in supercritical CO_2_ under varying temperatures and pressures.

(3) $$y_{2}=\frac{n_{REG}}{n_{1}+n_{REG}}$$

The following equation was applied to determine the solubility of *REG, S* (in g/L), in the supercritical CO_2_

(4) $$S=\frac{C_{REG}\left(\frac{g}{L}\right).V_{REG}\left(L\right)}{V_{1}\left(L\right)}$$

Eq. (4) can be reformulated as below:

(5) $$S=\frac{\rho M_{REG}}{M_{1}}\frac{y_{2}}{\left(1-y_{2}\right)}$$

In fact, Eq. (5) is another form of Eq. (4).

## 3 Modeling

Two different models, specifically 25 known density-based models, were used to look more closely at the *REG* solubility data that had been measured. The models were subsequently classified into three distinct categories according to their functional interdependence. Furthermore, the research utilized a model based on solid-liquid equilibrium (*SLE*) and the newly improved association models.

### 3.1 Semi-empirical models

Twenty-five density-based models were used to find correlations between the solubility data of *REG* in this study. These models were then categorized into three unique groups based on their functional interdependence. Table 3 provides an overview of density-based models utilized for the correlation of the solubility of the *REG* compound in ScCO_2_. The data utilized in the models comprises the solubility in mole fractions (*y*_2_), the density of ScCO_2_ (*ρ*_1_) was sourced from the National Institute of Science and Technology (NIST) database [96], and various reduced parameters including reduced density $\left(\rho_{r,1}=\frac{\rho_{1}}{\rho_{c,1}}\right)$, reduced temperature $\left(T_{r}=\frac{T}{T_{c,1}}\right)$, and pressure $\left(P_{r}=\frac{P}{P_{c,1}}\right)$ within the models of Alwi-Garlapati [79], Andonova-Garlapati [81], Sparks et al. [87], Reddy et al. [90], and Reddy-Garlapati [92]. The critical density of carbon dioxide (CO_2_) is denoted as *ρ*_*c,*1_ and has a value of 467.6 kg/m³. Similarly, the critical temperature of CO_2_, denoted as *T*_*c,*1_, is 304.12 K, and the critical pressure, denoted as *P*_*c,*1_, is 7.39 MPa. According to their paper, Bartle et al. (1991) [72] used a reference density (*ρ*_*ref*_ =-700 kg/m^3^) and a reference pressure (*P*_*ref*_ = 0.1 MPa).

**Table 3:** Density-based models are used to correlate the solubility of *REG* in ScCO_2_.

Models	Formula
Group I (the function of density, temperature, and pressure)	
Mendez - Teja [71]	*T* ln (*y*_2_*P*) = $a+b\rho_{1}+cT$
Bartle et al., [72]	ln $\left(\frac{y_{2}P}{P_{ref}}\right)$ = $a+b\left(\rho_{1}-\rho_{ref}\right)+\frac{c}{T}$
Jafari et al. [73],	ln *y*_2_ = $a+bP^{2}+cT^{2}+d\;\ln\;\rho_{1}$
Hozhabr et al. [74],	ln *y*_2_ = $a+\frac{b}{T}+c\frac{\rho_{1}}{T}-d\;\ln\;P$
Keshmiri et al.,[75]	ln *y*_2_ = $a+\frac{b}{T}+cP^{2}+\left(d+\frac{e}{T}\right)$
Khansary et al., [76]	ln *y*_2_ = $\frac{a}{T}+bP+c\frac{P^{2}}{T}+\left(d+e\;P\right)\;\ln\;\rho_{1}$
Jouyban et al. [77],	ln *y*_2_ = $a+bP+cP^{2}+dPT+e\frac{T}{P}+f\;\ln\;\rho_{1}$
Sodeifian et al. [17],	ln *y*_2_ = $a+b\frac{P^{2}}{T}+c\;\ln\left(\rho_{1}T\right)+d\left(\rho_{1}\;\ln\;\rho_{1}\right)+e\;P\;\ln\;T+f\frac{\rho_{1}}{T}$
Group II (the function of density and temperature)	
Kumar-Johnston [78]	ln *y*_2_ = $a+b\rho_{1}+\frac{c}{T}$
Alwi-Garlapati [79]	*y*_2_ = $\frac{1}{\left(\rho_{r,1}T_{r}\right)}\exp\left(a+\frac{b}{T_{r}}+c\rho_{r,1}\right)$
Chrastil [80]	ln *S* = $a+b\;\ln\left(\rho_{1}\right)+\frac{c}{T}$
Andonova-Garlapati [81]	*y*_2_ = ${a\;\rho }_{1}^{b}T_{r}^{c}$
Sung-Shim [82]	ln *y*_2_ = $\left(a+\frac{b}{T}\right)\ln\left(\rho_{1}\right)+\frac{c}{T}+d$
Garlapati-Madras [83]	ln *y*_2_ = $a+\left({b+c\;\rho }_{1}\right)\ln\;\rho_{1}+\frac{d}{T}+e\;\ln\;\rho_{1}$
Del Valle-Aguilera [84]	ln *y*_2_ = $a+\frac{b}{T}+\frac{c}{T^{2}}+d\;\ln\;\rho_{1}$
Bian et al.,[85]	ln *y*_2_ = $a+\frac{b}{T}+\frac{c\rho_{1}}{T}+\left({d+e\;\rho }_{1}\right)\ln\;\rho_{1}$
Adachi-Lu [86]	ln *y*_2_ = $a+\left({b+c\;\ln\;\rho }_{1}+d\;\ln\;{\;\rho_{1}}^{2}\right)\ln\;\rho_{1}+\frac{e}{T}$
Sparks et al., [87]	*S*_*2*_ = $\rho_{r,1}^{\left(a+b\rho_{1}+\frac{c}{\ln\;T}\right)}\;\exp\left(d+\frac{e}{T_{r}}+\frac{f}{T_{r,1}^{2}}\right)$
Si-Moussa et al., [88]	ln *y*_2_ = $a+b\rho_{1}+c{\rho_{1}}^{2}+d\rho_{1}T+e\frac{T}{\rho_{1}}+f\;\ln\;\rho_{1}$
Belghait et al., [89]	ln *y*_2_ = $a+b\rho_{1}+c{\rho_{1}}^{2}+d\rho_{1}T+eT+fT^{2}+g\;\ln\;\rho_{1}+\frac{h}{T}$
Group III (the function of pressure and temperature)	
Reddy et al., [90]	*y*_2_ = (*a* + *b P*_*r*_) *T*^2^ + (*c* + *d P*_*r*_) *T*_*r*_ + *e*
Mitra-Wilson [91]	ln *S*_2_ = *a* ln *P* + *b T* + *c P T* + *d*^*P*^ + *e*
Reddy-Garlapati [92]	*y*_2_ = (*a* + *b P*_*r*_ + *c P*^2^) *T*_*r*_ + (*d* + *e P*_*r*_ + *f P_r_*^2^)
Gordillo et al., [93]	ln *y*_2_ = *a* + *bP* + *cP*^2^ + *dPT* + *eT* + *f T*^2^
Yu et al., [94]	*y*_2_ = *a* + *b P* + *c P*^2^ + *d P T* (1 − *y*_2_) + *e T* + *f T*^2^

### 3.2 Solid liquid equilibrium models (SLE)

The solubility of solids in ScCO_2_ has been associated with molecular association citation [80]. It is hypothesized that each molecule of the solid solute (*A*) forms an association with one or more molecules of ScCO_2_ (*B*).

(6) *A* + *κB* ⇔ *AB*_*κ*_

The expression for the constant in Eq. (6) is given by considering the arrangement of the molecules as

(7) $$K_{f}=\frac{{\left(\frac{{\hat{f}}_{AB_{K}}}{f_{AB_{K}}^{*}}\right)}_{SCP}}{{\left(\frac{{\hat{f}}_{A}}{f_{A}^{*}}\right)}_{S}{\left(\frac{{\hat{f}}_{B}}{f_{B}^{*}}\right)}_{SCP}}$$

In Eq. (7) *SCP* is the supercritical phase, *S* denotes solute phase, and $\hat{f}$ is fugacity.

The solubility of the solute is given by *x*_2_ = *x*_*ABκ*_, and the fugacity of the

complex is denoted by the activity coefficient as the solute molecule is primarily present in the associated form in SCP.

(8) $${\hat{f}}_{AB_{K}}^{SCP}=x_{2}\gamma_{2}f_{AB_{K}}^{l}$$

The solid solute fugacity is

(9) $${\hat{f}}_{A}=P_{A}^{sub}\;\exp\left(\frac{V_{A}\left(P-P_{A}^{sub}\right)}{RT}\right)$$

The reference fugacity (*f* ∗) and the SCP fugacity were given by

(10) $${\hat{f}}_{B}=x_{B}{\hat{\emptyset }}_{B}P$$

(11) $$f_{j}^{*}=\emptyset_{j}^{*}P^{*}$$

By substituting Eqs. (8)–(11) into (7) for *j* = *A, B, AB*_*κ*_, and *x*_2_ = *y*

(12) $$K_{f}=\frac{\left(y\emptyset_{AB_{K}}/\emptyset_{AB_{K}}^{*}P^{*}\right)}{{\left(P_{A}^{sub}\;\exp\left(\frac{V_{A}\left(P-P_{A}^{sub}\right)}{RT}\right)/\emptyset_{2}^{*}P^{*}\right)\left(y_{B}{\hat{\emptyset }}_{B}P/\emptyset_{B}^{*}P^{*}\right)}^{K}}$$

Eq. (12) can be modified as follows

$$\ln\left(K_{f}\right)=\ln\left(y\right)+\ln\left(\frac{\emptyset_{AB_{K}}}{\emptyset_{AB_{K}}^{*}}\right)+\ln\left(\frac{P}{P^{*}}\right)+\ln\left(\emptyset_{A}^{*}\right)-\ln\left(\frac{P_{A}^{sub}}{P^{*}}\right)-$$

(13) $$\frac{V_{A}\left(P-P_{A}^{sub}\right)}{RT}-K\;\ln\left(y_{B}\right)-K\;\ln\left(\frac{{\hat{\emptyset }}_{B}}{\emptyset_{B}}\right)-K\;\ln\left(\frac{P}{P^{*}}\right)$$

The constant denoted as (*K*_*f*_), can be mathematically represented as

(14) $$\ln\left(K_{f}\right)=\frac{\Delta H_{s}}{RT}+q_{s}$$

In Eq. (14), *q*_*s*_ is a constant. The term *V*_*A*_*P/RT* is modified to be $\frac{ZV_{A\rho_{1}}}{M_{w}}$. Thus, Eq.

(13) can be rewritten as

$$\ln\left(y\right)-K\;\ln\left(y_{B}\right)+\left(1-K\right)\;\ln\left(\frac{P}{P^{*}}\right)=\ln\left(\frac{\emptyset_{AB_{K}}}{\emptyset_{AB_{K}}^{*}}\right)+\ln\;\left(\emptyset_{A}^{*}\right)-\ln\left(P_{A}^{sub}\right)+$$

(15) $$\ln\left(P^{*}\right)-\frac{ZV_{A\rho_{1}}}{M_{w}}+\frac{V_{A}P_{A}^{sub}}{RT}-K\;\ln\left(\frac{\emptyset_{B}}{\emptyset_{B}^{*}}\right)+\frac{\Delta H_{s}}{RT}+q_{s}$$

The sublimation pressure of the solute $\left(P_{A}^{sub}\right)$ can be expressed as

(16) $$\ln\left(P_{A}^{sub}\right)=a_{1}-\frac{b_{1}}{T}$$

Eq. (16), contains parameters *a*_1_ and *b*_1_.

#### 3.2.1 Rajasekhar-Madras’ model

In 2010, Rajasekhar-Madras et al. [95], introduced a model grounded in association theory that incorporated four modifiable parameters:

In Eq. (15), the term, *V*_2_*P*^*sub*^*/RT* can be neglected because molar volume and sublimation pressure are very small. Therefore, Eq. (15) becomes.

$$\ln\left(y\right)-K\;\ln\left(y_{B}\right)+\left(1-K\right)\;\ln\left(\frac{P}{P^{*}}\right)=\frac{D_{1}}{T}+D_{2}\rho_{1}+D_{3}$$

(17) $$For\;D_{1}=\frac{\Delta H_{s}}{R}-b_{1},D_{2}=\frac{ZV_{A\rho_{1}}}{M_{w}},and\;D_{3}=\ln\;\left(\left(\emptyset_{A}^{*}\right)\left(\frac{\emptyset_{AB_{K}}}{\emptyset_{AB_{K}}^{*}}\right)/{\left(\frac{{\hat{\emptyset }}_{B}}{\emptyset_{B}}\right)}^{K}\right)-\ln\left(P^{*}\right)+\;q_{s}+a_{1}$$

thus Eq. (17) can be written as follows:

(18) $$y_{2}={\left(y_{B}\right)}^{K}{\left(\frac{P}{P^{*}}\right)}^{\left(K-1\right)}\;\exp\left(\frac{D_{1}}{T}+D_{2}\rho_{1}+D_{3}\right)$$

In this equation, *ρ*_1_ denotes the ScCO_2_ density in mol/mL, and K is the association number. *D*_1_ through *D*_3_ are model parameters. Since drugs do not dissolve well in ScCO_2_, we can assume that $y_{B}$ is equal to one for binary systems. Therefore, Eq. (18) becomes

(19) $$y_{2}={\left(\frac{P}{P^{*}}\right)}^{\left(K-1\right)}\;\exp\left(\frac{D_{1}}{T}+D_{2}\rho_{1}+D_{3}\right)$$

The standard pressure, *P* ∗, is defined as 0.1 MPa.

#### 3.2.2 New modified association theory models

In terms of temperature and density dependence, the solubility is directly related to the reduced temperature, $T_{r}\left(T_{r}=\frac{T}{T_{c,1}}\right)$ and the reduced density, $\rho_{r,1}=\frac{\rho_{1}}{\rho_{c,1}}$ , Eq. (19) can be modified to be

(20) $$y_{2}={\left(\frac{P}{P^{*}}\right)}^{\left(K-1\right)}\;\exp\left(\frac{A_{1}}{T_{r,1}}+A_{2}\rho_{r,2}+A_{3}\right)$$

where the critical temperature of CO_2_, denoted as *T*_*c,*1_, is 304.12 K, *A*_1_ − *A*_3_ are parameters, and *P* ^∗^ is standard pressure (=0.1 MPa). Moreover, the Eq.(19) can be modified and becomes

(21) $$y_{2}={\left(\frac{P}{P^{*}}\right)}^{\left(K-1\right)}\;\exp\left(\frac{C_{1}}{T}+C_{2}\rho_{1}+C_{3}P+C_{4}\right)$$

by assumptions for the solubility equation.

• Linear Dependency on Parameters: The solubility is influenced linearly by the terms inside the exponential function (e.g., density, temperature or pressure) will result in proportional changes in solubility.
• Ideal Gas Behavior: The term ${\left(\frac{P}{P^{*}}\right)}^{\left(K-1\right)}$ suggests an ideal gas-like behavior. This assumes that the solute behaves like an ideal gas in terms of its response to pressure changes.
• Standard Pressure Independence: The standard pressure *P*^∗^ is set at 0.1 MPa and does not influence the solubility beyond its presence in the equation.

In Eq. (21), *κ* is the association number, *C*_1_ - *C*_4_ are parameters, *P*^∗^ is standard pressure, *P* is system pressure, and *ρ*_1_ is density of carbon dioxide (CO_2_).

### 3.3 Statistical techniques

Statistical methods were used to test how well the above models could correlate by comparing the predicted solubility values from each model to those found in the experiments. The average absolute relative deviation (*AARD*%), (in Eq. (22)), correlation coefficient (*R*^2^), Eq. (23), adjusted correlation coefficient (*R*_*adj*_), (see Eq. (24)), sum of squares due to error (*SSE*), Eq. (25), root mean square deviation (*RMSE*), (in Eq. (26)), were utilized to optimize the adjustable parameters of semi-empirical models.

(22) $$AARD\left(\%\right)=\frac{100}{N_{i}}\sum_{i=1}^{N_{i}}\frac{\left|y_{2}^{cal}-y_{2}^{\exp}\right|}{y_{2}^{\exp}}\;$$

(23) $$R^{2}=1-\frac{\sum_{i=1}^{N_{i}}{\left(y_{2}^{\exp}-y_{2}^{cal}\right)}^{2}}{\sum_{i=1}^{N_{i}}{\left(y_{2}^{\bar{\exp}}-y_{2}^{cal}\right)}^{2}}$$

(24) $$R_{adj}=\sqrt{\left|R^{2}-\left(Q\left(1-R^{2}\right)/\left(N-Q-1\right)\right)\right|}$$

(25) $$SSE=\left[\sum_{i=1}^{N_{i}}{\left(y_{2}^{\exp}-y_{2}^{cal}\right)}^{2}\right]$$

(26) $$RMSE={\left[\sum_{i=1}^{N_{i}}{\left(y_{2}^{\exp}-y_{2}^{cal}\right)}^{2}\right]}^{\frac{1}{2}}$$

In Eqs. (22), (23), (24), (25), (26), $y_{2}^{cal}$ and $y_{2}^{\exp}$ denote the mole fraction of *REG* solubility

values computed and experimental, respectively. The variable *N*_*i*_ represents the quantity of data points, whereas the variable *Q* denotes the number of independent variables present in each model used.

## 4 Results and discussion

### 4.1 Experimental results

In order to establish the reliability and standardization of the approach, the apparatus and procedure underwent prior validation. This validation involved an examination of the solubility of *α-Tocopherol* and *Naphthalene* under varying temperatures and pressures. The obtained results were then compared with existing data in the literature [16]. In accordance with the aforementioned experimental methodology, the solubility of *REG* in supercritical carbon dioxide (ScCO_2_) was determined across a temperature range of 308–338 K and pressures ranging from 120 to 270 bar. In order to enhance the precision of the measurements, the experiment was conducted in triplicate. Each presented datum is the mean value derived from three separate and independent experimental mental measurements. The data *REG* solubility obtained, which encompass the equilibrium mole fraction and solubility estimated by equations (1), (2), (3), (4), have been compiled and shown in Table 2. It is evident that the equilibrium mole fraction exhibited both minimum and maximum values at a temperature of 338 K. Specifically, the minimum value was determined to be 3.06 × 10^−7^, while the maximum value was found to be 6.44 × 10^−6^. Figure

**Fig. 2:**
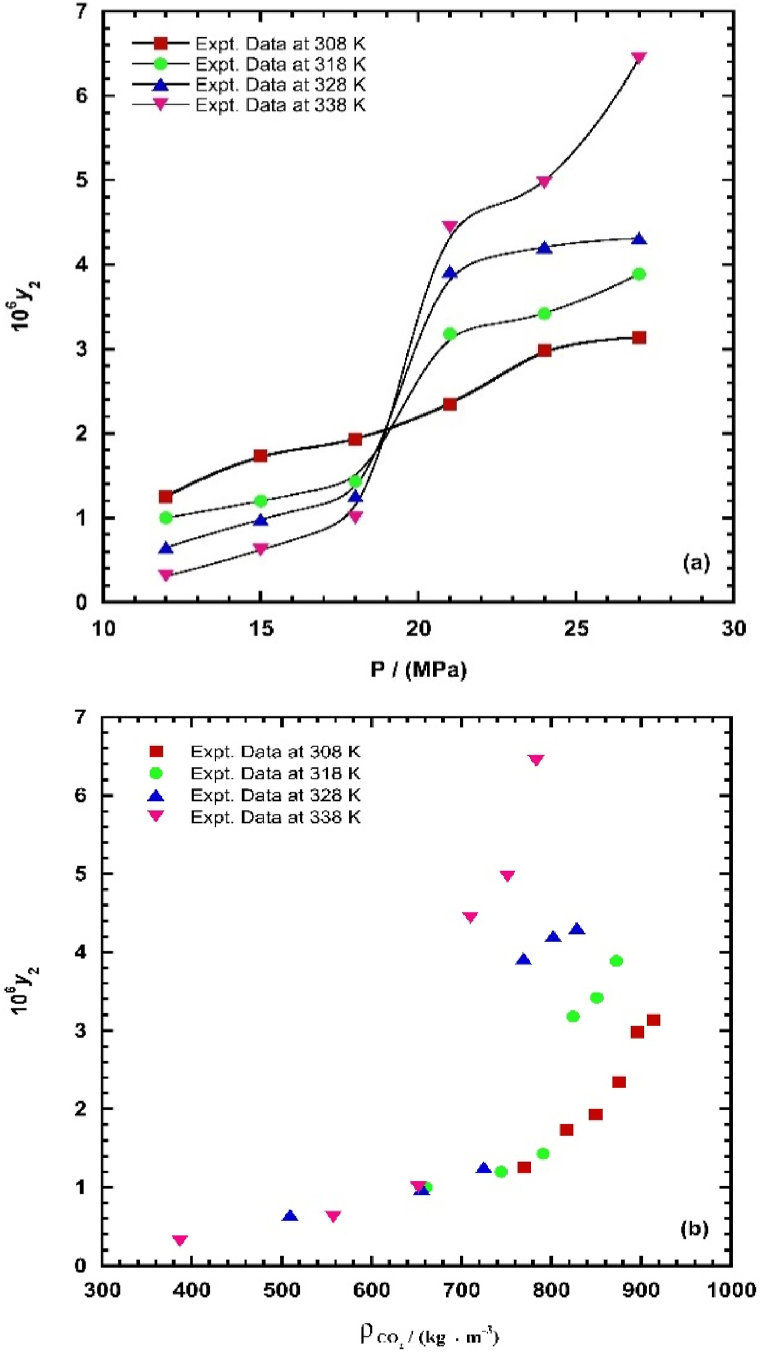
*REG* solubility in ScCO_2_ against (a) pressure, and (b) density of ScCO_2_.

2 illustrates the equilibrium mole fractions of *REG* as a function of CO_2_ density and pressure, as determined under the specific temperature of the system. In order to determine the necessary density (*ρ*) of supercritical carbon dioxide (ScCO_2_) at each specific position, the data was sourced from the National Insti-tute of Science and Technology (NIST) database [96]. As demonstrated, there is a positive correlation between the density of CO_2_ and the equilibrium mole fraction values. Additionally, it can be shown from Fig. 2 that the solubility isotherms exhibit an increasing tendency as the pressure of the system increases. Moreover, it is evident that the isotherms meet at the pressure range of 18–21 MPa, resulting in the emergence of a crossover pressure point. This occurrence is sometimes ascribed to the point at which the powers of two contending fac-tors, specifically the vapor pressure of a solid and the density of carbon dioxide, become equivalent. The impact of temperature on the solubility of solid solutes exhibits a contradictory behavior when subjected to an isobaric transformation occurring before and after the crossover point, as depicted in Fig. 2. The observed behavior of temperature in relation to solubility at pressures of 12–18 MPa suggests that the primary factor influencing solubility is the drop in CO_2_ density. Conversely, at pressures of 21–27 MPa, the increasing behavior of temperature indicates that the increase in the solute's vapor pressure is the dominant factor [100]. The occurrence of the crossover phenomena and the dualistic influence of temperature have been extensively documented in the scientific literature on the solubility of several chemicals in supercritical carbon dioxide (ScCO_2_) [[8], [11], [101], [102], [103]].

**Fig. 3:**
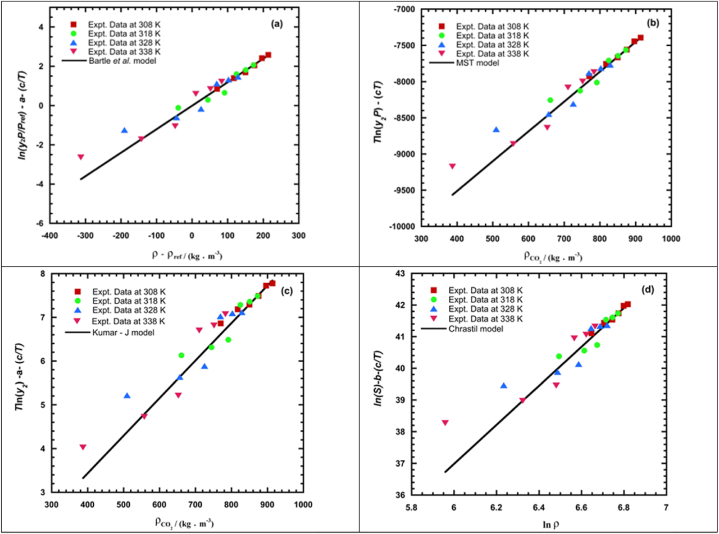
The experimental data of *REG* exhibit self-consistency when compared to various empirical models, particularly (a) Bartle et al. [72], (b) Mendez-Teja [71], (c) Kumar-Johnston [78], and (d) Chrastil [80].

In Fig. 3, the self-consistency test of experimental REG solubility data employed the models Chrastil [80], Kumar-Johnston [78], Bartle et al. [72], and Mendez-Teja [71]. Bartle et al., and Mendez–Teja demonstrated good self-consistency, as depicted in Fig. 3. Self-consistency tests were conducted on the solubility data to demonstrate the degree to which the empirical models could successfully correlate the solubility values. Subjecting the empirical density based models to self-consistency tests, the results proved the reliability of using the model parameters for estimating the solubility. In other words, wherein all data points at different isotherms falls onto a single straight line, indicating the self-consistency test for the solubility of REG in ScCO_2_ at different temperatures.

Analogous techniques were applied for Galantamine [59], Clonazepam [103], Lansoprazole [10], and Esomeprazole [62]. Finally, the total (Δ*H*_tot_-=-41.16 kJ/mol), vaporization (Δ*H*_vap_-=-60.04 kJ/mol), and solvation (Δ*H*_sol_-=-18.88 kJ/mol) enthalpies for the *REG*-ScCO_2_ binary system were estimated as part of this work. More detail of enthalpies of the *REG* can be seen in Table 4.

**Table 4:** Approximated the total heat, the vaporization heat and the solvation heat for *REG* drug solubility.

System	Δ*H*_tot_/(kJ*/*mol)^a^	Δ*H*_tot_/(kJ*/*mol)^b^	Δ*H*_vap_/(kJ*/*mol)^c^	Δ*H*_sol_/(kJ*/*mol)^d^
*4*	41.17	41.14	60.04	18.88

a The examination is based on the Chrastil model [80].

b The examination is based on Kumar-Johnston model [78].

c The examination is based on Bartle et al., model [72].

d The evaluation is predicated on the disparity between Bartle et al. [72], and ((Δ*H*_tot_)^*a*^+ (Δ*H*_tot_)^*b*^)/2.

### 4.2 Modeling for REG solubility

As previously stated, the recent work employed two methodologies to establish a correlation between the experimental data. These methodologies consisted of the utilization of a collection of density based models and a collection of solid-liquid equilibrium models. Subsequently, in order to assess the predictive capacity, the statistical criteria were employed to calculate the correlation findings for each model. Additionally, the predicted isotherms were graphically juxtaposed with the experimental data points for visual comparison.

#### 4.2.1 Semi-empirical models

A comprehensive set of 25 density-based models was utilized in this research to ascertain correlations among the solubility data of *REG*. On the basis of their functional interdependence, these models were subsequently classified into three distinct groups. An overview of the density-based models employed to correlate the solubility of the *REG* compound in supercritical carbon dioxide (ScCO_2_) is presented in Table 3. The results of correlation parameters and statistical analysis (*SSE*, *R*^2^, *R*_*adj*_, *RMSE*, and *AARD*%) for *REG* solubility in ScCO_2_ are summarized in Table 5. The best model to correlate the solubility of *REG* in ScCO_2_ was presented by Gordillo et al. [93], (*AARD*-=-13.2-%) and Reddy et al. [90], (*AARD*-=-13.5-%) out of 25 density-based models (see Table 5). The models are classified into group III (as a function of pressure and temperature). Gordillo et al. [93], (*AARD*-=-13.2-%) provided the logarithm of the mole fraction solubility of *REG* has a linear dependence on the pressure and temperature with six adjustable parameters. Moreover, Reddy et al. [90], provided the logarithm of the mole fraction solubility of *REG* has a linear dependence on the reduced pressure (*P*_*r*_) and reduced temperature. In addition, Gordillo et al. [93], provided the logarithm of the mole fraction solubility of *REG* has a linear dependence on the pressure and temperature with six adjustable parameters. Moreover, Reddy et al. [90], also present the logarithm of the mole fraction solubility of *REG* with respect to the reduced pressure (*P*_*r*_) and reduced temperature (*T*_*r*_) with five adjustable parameters.In general, adjustable parameters in EoSs or other models may be determined by different methods such as nonlinear regression methods [[104], [105]].

**Table 5:** Correlation parameters and *AARD*% for *REG* solubility in ScCO_2_

Models	Parameters								SSE	$R^{2}$	$R_{adj}$	RMSE	AARD
	a	b	c	d	e	f	g	h	×)			×)	%
Group I (a function of density, temperature, and pressure)													
Mendez - Teja [71]	-11229	4.13	1713						4.82	0.898	0.893	4.68	22
Bartle *et al.*, [72]	13.79	0.0120	-7221						5.17	0.879	0.873	4.85	22.1
Jafari *et al.*, [73]	-71.74	-1.38$\times 10^{-6}$	9.48$\times 10^{-5}$	7.359					5.1	0.916	0.913	4.81	22.9
Hozhabr *et al.*, [74]	24.24	-8.633	4.191	3.106					6.25	0.837	0.829	5.33	21.2
Keshmiri *et al.*, [75]	12.64	-13819	1.53$\times 10^{-5}$	-3.565	1943.3				10.4	0.633	0.616	6.87	21.1
Khansary *et al.*, [76]	-6058	-0.259	-9.29$\times 10^{-3}$	0.806	0.04				7.93	0.779	0.769	6	22.7
Jouyban *et al.*, [77]	-68.99	1.368	-0.024	3.62$\times 10^{-4}$	0.558	4			5	0.875	0.869	4.77	16.7
Sodeifian *et al.*, [17]	-50.06	9.08$\times 10^{-3}$	2.702	-4.25$\times 10^{-4}$	-6.31$\times 10^{-4}$	240.9			15.7	0.421	0.395	8.44	20.6
***Average:***													**21.2**
Group II (a function of density and temperature)													
Kumar – Johnston [78]	-4.386	8.59$\times 10^{-3}$	-4947.8						5.90	0.842	0.834	5.18	21.4
Alwi-Garlapati [79]	3.877	-17.32	4.639						6.06	0.836	0.828	5.25	21.2
Chrastil [80]	7.165	-38.62	-4952.4						5.08	0.879	0.874	4.81	23
Andonova-Garlapati [81]	1.08$\times 10^{-5}$	1.644	-7.638						12.8	0.303	0.271	7.65	27.1
Sung-Shim [82]	7.966	-6.23.1	-6.30.9	-51.14					5.19	0.874	0.868	4.86	23.1
Garlapati-Madras [83]	-48.25	-14.75	1.88$\times 10^{-3}$	17025	0.033				4.99	0.896	0.891	4.76	19.2
Del Valle-Aguilera [84]	10.30	-38327	5.20$\times 10^{6}$	6.844					4.99	0.896	0.891	4.76	22.1
Bian *et al.*, [85]	-13.32	5834	-12.85	-3.371	6.89$\times 10^{-3}$				7.41	0.770	0.760	5.81	17.2
Adachi-Lu [86]	-3.258	1.73$\times 10^{-3}$	-1.94$\times 10^{-8}$	15.39	-5056.8				6.43	0.824	0.816	5.11	20
Sparks *et al.*, [87]	-3.046	6.954	-0.937	211.08	-111.1	-123.2			6.43	0.790	0.781	5.11	23.2
Si-Moussa *et al.*, [88]	-135.7	-0.02.38	4.1$\times 10^{-6}$	3.41$\times 10^{-5}$	17.36	18.33			5.92	0.842	0.835	5.19	19.5
Belghait *et al.*, [89]	287.08	-0.31	9.07$\times 10^{-5}$	1.88$\times 10^{-4}$	-2.818	0.0033	89.34	-61578	5.70	0.892	0.887	5.09	22.6
***Average:***													**21.6**
Group III (a function of pressure and temperature)													
Reddy *et al.*, [90]	-40.98	9.451	54.64	-9.204	-27.33				9.64	0.809	0.801	6.62	13.5
Mitra-Wilson [91]	3.313	-0.122	2.84$\times 10^{-4}$	-31	9.57				9.05	0.850	0.801	6.41	16.2
Reddy-Garlapati [92]	-1.19$\times 10^{-5}$	6.63$\times 10^{-7}$	4.94$\times 10^{-7}$	1.47$\times 10^{-5}$	-2.66$\times 10^{-6}$	1.8$\times 10^{-7}$			12.5	0.681	0.667	7.54	16.5
Gordillo *et al.*, [93]	10.38	-1.298	-2.45$\times 10^{-3}$	4.72$\times 10^{-3}$	-0.064	-6.15$\times 10^{-5}$			8.38	0.843	0.836	6.17	13.2
Yu *et al.*, [94]	2.56$\times 10^{-5}$	-2.19$\times 10^{-7}$	1.56$\times 10^{-10}$	5.72$\times 10^{-10}$	-3.82$\times 10^{-8}$	-1.02$\times 10^{-10}$			8.47	0.835	0.828	6.2	17.2
***Average:***													**15.3**

#### 4.2.2 Solid-liquid equilibrium models

**Fig. 4:**
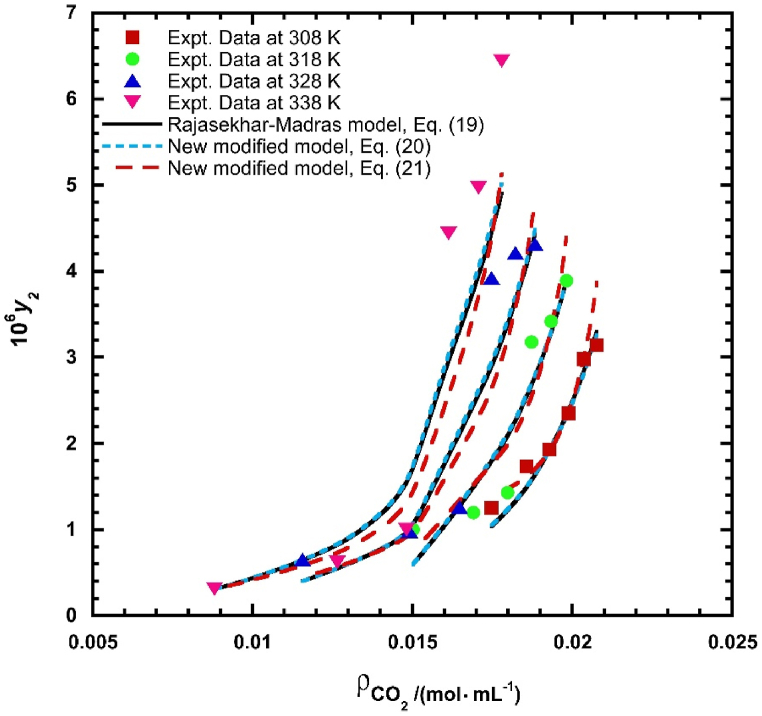
The solubility of *REG* in ScCO_2_ at different temperatures through the utilization of association models visualized.

According to the explanation in section 3.2.2. Three solid-liquid equilibrium models were used to find correlations between the solubility data of *REG*. These models were then categorized as association theory models, specifically Rajasekhar-Madras [95], and two newly modified association theory models. supporting information (S2) provides an overview of Rajasekhar-Madras [95] utilized for the correlation of the solubility of *REG*. The two newly modified association theory models successfully correlated solute solubility of 15 drug compounds in supercritical carbon dioxide (ScCO_2_). The solubility information of the compounds considered to test the models is presented in supporting information (S1). The correlating capability of the new models was assessed using some various statistical parameters (*SSE*, *R*^2^, *R*_*adj*_, *RMSE*, and *AARD*%). Furthermore, Table 6, Table 7 summarize correlations parameters and statistical criterion of two new modified association theory models (see Eqs. (20), (21)), respectively. The illustration of all association models at different temperatures can be seen in Fig. 4. It is evident that all association theory models exhibit a strong correlation with experimental solubility data. Consequently, it is strongly advised that the new association theory models be utilized to correlate drug solubility in ScCO_2_.

**Table 6:** Correlation parameters and statistical analysis (*SSE*, *R*^2^, *R*_*adj*_, *RMSE*, and *AARD*%) of new modified model (see Eq. (20)) for each drug compound considered.

Compound	*κ*	*B*_1_	*B*_2_	*B*_3_	*SSE-*( × 10^11^)	*R*^2^	*R*_*adj*_	*RMSE-*( × 10^6^)	*AARD*%
*Clonazepam*	0.900	−14.59	3.095	−1.755	8.734	0.990	0.989	1.833	4.45
*4-Methyl-N-phenylacetanilide*	0.495	−33.63	3.992	18.14	454.0	0.978	0.977	15.46	6.89
*4-Methylbenzoic acid*	1.050	−1.241	−0.013	−7.730	0.022	1.000	1.000	0.117	0.05
*Benzoin*	0.559	−20.93	3.471	6.851	237.5	0.987	0.987	11.82	5.63
*Busulfan*	0.724	−20.64	3.558	5.414	1433	0.986	0.985	21.86	7.27
*Clofenamic acid*	0.004	−17.93	4.978	5.246	69105	0.987	0.986	177.2	3.79
*Climbazole*	0.004	−17.93	4.978	5.246	69105	0.987	0.986	177.2	3.79
*Clofibric acid*	−0.052	−6955	402.5	12.70	930.5	0.991	0.990	22.13	3.45
*Fenofibrate*	0.001	−21.89	4.921	9.932	80569	0.990	0.989	205.9	4.28
*Gemfibrozil*	0.001	−26.77	5.525	12.91	17925	0.994	0.993	97.13	3.67
*Lamotrigine*	−0.001	−19.71	3.609	2.416	0.055	0.992	0.992	0.127	3.63
*Prazosin hydrochloride*	2.165	−4.634	−0.289	−9.055	13.66	0.968	0.966	2.492	4.81
*Pholcodine*	3.972	−5.778	−0.612	−15.89	1.126	0.928	0.924	0.715	17.2
*Dasatinib monohydrate*	1.292	−6.522	0.973	−4.296	2126	0.927	0.924	31.09	6.86
*Regorafenib monohydrate*	1.472	−12.67	2.291	−6.582	0.417	0.898	0.894	0.435	20.1
**Mean**									**6.39**

**Table 7:** Correlation parameters and statistical analysis (*SSE*, *R*^2^, *R*_*adj*_, *RMSE*, and *AARD*%) of new modified association model (see Eq. (21)) for each drug compound considered.

Compound	*κ*	*C*_1_	*C*_2_	*C*_3_	*C*_4_	*SSE* ( × 10^11^)	*R*^2^	*R*_*adj*_	*RMSE* ( × 10^6^)	*AARD* %
*Clonazepam*	1.238	−4219	296.6	−0.012	−3.559	1.066	0.987	0.986	2.025	4.93
*4-Methyl-N-phenylacetanilide*	2.536	−11050	441.5	−0.149	13.13	57.81	0.968	0.966	17.44	6.28
*4-Methylbenzoic acid*	1.014	−393	−0.488	0.002	−7.642	0.0003	1.000	1.000	0.045	0.02
*Benzoin*	2.239	−5569	308.0	−0.080	−1.338	13.43	0.993	0.992	8.889	4.07
*Busulfan*	1.391	−5394	265.6	−0.009	1.490	182.0	0.981	0.980	24.63	7.48
*Clofenamic acid*	0.432	−7910	414.1	−0.013	9.109	0.207	0.988	0.988	0.970	4.42
*Climbazole*	1.934	−3072	366.7	−0.054	−6.901	4628	0.990	0.990	145.0	3.89
*Clofibric acid*	7.904	−3830	245.1	−0.404	−28.26	845.2	0.922	0.918	66.70	12.2
*Fenofibrate*	3.583	−5782	441.9	−0.203	−4.798	18491	0.975	0.974	312.0	6.19
*Gemfibrozil*	7.088	−6507	441.6	−0.379	−18.14	12242	0.955	0.953	253.8	11.3
*Lamotrigine*	0.964	−5258	304.4	−0.024	−1.498	0.005	0.993	0.993	0.119	4.13
*Prazosin hydrochloride*	3.098	−1351	−31.36	−0.049	−15.45	1.057	0.974	0.972	2.191	4.50
*Pholcodine*	6.514	−2833	−26.63	−0.161	−29.19	0.132	0.912	0.908	0.776	13.5
*Dasatinib monohydrate*	3.830	−1042	36.98	−0.106	−18.01	262.6	0.922	0.918	34.55	7.70
*Regorafenib monohydrate*	−1.657	−3611	255.30	0.156	4.61	7.358	0.832	0.824	0.578	18.8
**Mean**										**7.29**

## 5 Conclusion

This investigation determined the solubility of *Regorafenib monohydrate* (*REG*) in ScCO_2_, for the first time. Utilizing a static method, the experimental conditions encompassed isotherms spanning a temperature range of 308–338 K, along with six distinct pressures ranging from 120 to 270 bar. The solubilities of *REG* in ScCO_2_ varied between 3.06-×-10^−7^ and 6.44-×-10^−6^. Among 25 density models, Gordillo et al., (*AARD*-=-13.2 %) and Reddy et al., (*AARD*-=-13.5-%) models effectively captured and correlated the *REG's* solubility data in ScCO_2_. From the model constants of Chrastil, Kumar-Johnston, and Bartle et al., the enthalpies of the ScCO_2_ *REG* mixture was determined. Three association models were applied to the solubility data. The model results indicate that all them reasonably fit the data. Given the order of magnitude of *REG's* solubility in ScCO_2_, supercritical anti-solvent techniques may be deemed suitable for the production of nanoscale particles of this pharmaceutical compound.

## Data availability

Data will be made available on request.

## CRediT authorship contribution statement

**Gholamhossein Sodeifian:** Writing – review & editing, Supervision, Resources, Project administration, Methodology, Investigation, Funding acquisition. **Ratna Surya Alwi:** Writing – original draft, Software, Methodology, Investigation, Formal analysis. **Fatemeh Sodeifian:** Resources, Funding acquisition. **Solmaz Amraee:** Resources, Data curation. **Mohammadreza Rashidi-Nooshabadi:** Formal analysis. **Fariba Razmimanesh:** Methodology.

## Declaration of competing interest

The authors declare that they have no known competing financial interests or personal relationships that could have appeared to influence the work reported in this paper.

## Nomenclature

*a* – *f*: density based models parameters (Table 3)
*a*_1_, *b*_1_: parameters of Eq. (16) (F-1) – (F-16) experimental parts
*A*_1_ − *A*_3_: parameters of Eq. (20)
*AARD*%: average absolute relative deviation (in Eq. (22))
*C*_1_ - *C*_4_: adjustable parameters of Eq. (21)
*C*_*REG*_: REG's concentration in collection vial (g/L), Eq. (1), and Eq. (4)
*D*_1_ - *D*_3_: model parameters of Eq. (18)
DMSO: dimethyl sulfoxide, Table 1
*f* ∗: the reference fugacity Eqs. (7)–(9)
FDA: food and drug administration
*f*^^^: fugacity, Eq. (7)
Δ*H*_tot_: the total reaction of REG drug (Table 4)
Δ*H*_vap_: the vaporization of REG drug (Table 4)
Δ*H*_sol_: the solvation heat of *REG* drug (Table 4)
*M*_1_: molar masses of CO_2_, Eq. (2)
*M*_*REG*_: molar masses of REG, Eq. (4)
*n*_*REG*_: the REG's moles Eq. (1), and Eq. (3)
*n*_1_: CO_2_ moles, Eq. (2), and Eq. (3)
*P*_*c,*1_: the critical pressure of CO_2_ (Table 3)
*P*_*r*_: reduced pressure (Table 3)
*P* ∗: the standard pressure (=0.1 MPa)
$P_{A}^{\text{sub}}$: the sublimation pressure, Eq. (16)
*REG*: Regorafenib monohydrate
*S*: solute phase, Eq. (7)
*SCP*: the supercritical phase, Eq. (7)
*SLE*: solid-liquid equilibrium, Table 3
*T*_*c,*1_: the critical temperature of CO_2_, Table 3
*T*_*r*_: reduced temperature, Table 3
*V*_*REG*_(*L*): the collection vial, Eq. (1), (4)
*V*_1_: the sampling loop, Eq. (2), (4)

**Greek symbols**
*ρ*_1_: density of CO_2_ (kg*/*m^3^, mol/mL)
*φ*: fugacity coefficient
*κ*: the association number Eq. (19), (20)
