# Supplementary material for: Determination of Regorafenib monohydrate (colorectal anticancer drug) solubility in supercritical CO2: Experimental and thermodynamic modeling
Source: Heliyon. 2024 Apr 15;10(8):e29049. doi: 10.1016/j.heliyon.2024.e29049 (PMC11052913; doi:10.1016/j.heliyon.2024.e29049)
Supplement: Multimedia component 1 [file mmc1.doc]

**Determination of *Regorafenib monohydrate (colorectal anticancer drug)* solubility in supercritical CO_2_: Experimental and thermodynamic modeling﻿**

**Gholamhossein Sodeifian^a,b,c,∗^, Ratna Surya Alwi^d,e^, Fatemeh Sodeifian^f^, Solmaz Amraee^a,b,c^, Mohammadreza Rashidi-Nooshabadi^g^, Fariba Razmimanesh^a,b,c^**

*^a^Department of Chemical Engineering, Faculty of Engineering, University of Kashan, Postal Code: 87317-53153, Kashan, Iran.*

*^b^Laboratory of Supercritical Fluids and Nanotechnology, University of Kashan, Postal Code: 87317-53153, Kashan, Iran.*

*^c^Modeling and Simulation Centre, Faculty of Engineering, University of Kashan, Postal Code: 87317-53153, Kashan, Iran.*

*^d^Research Centre for Computing, National Research and Innovation Agency (BRIN), Jl.*

*Raya Jakarta-Bogor KM 46 Cibinong, Indonesia*

*^e^Collaboration Research Center for Precision Oncology based Omics (PrOmics), Indonesia*

*^f^School of Medicine, Shahid Beheshti of Medical Sciences, Postal Code:*

*^g^Department of Pharmacology, School of Medicine, Kashan University*

*of Medical Sciences, Postal Code: 87159-88141 Kashan, Iran*

*Corresponding author. Tel.: +983155912406; fax: +98315591*2*424.

E-mail address: **sodeifian@kashanu.ac.ir (**G. Sodeifian**)**

Table S1: The solubility data of the compounds under consideration is provided.

Compound Structure N T(K) P Mole fraction Ref.


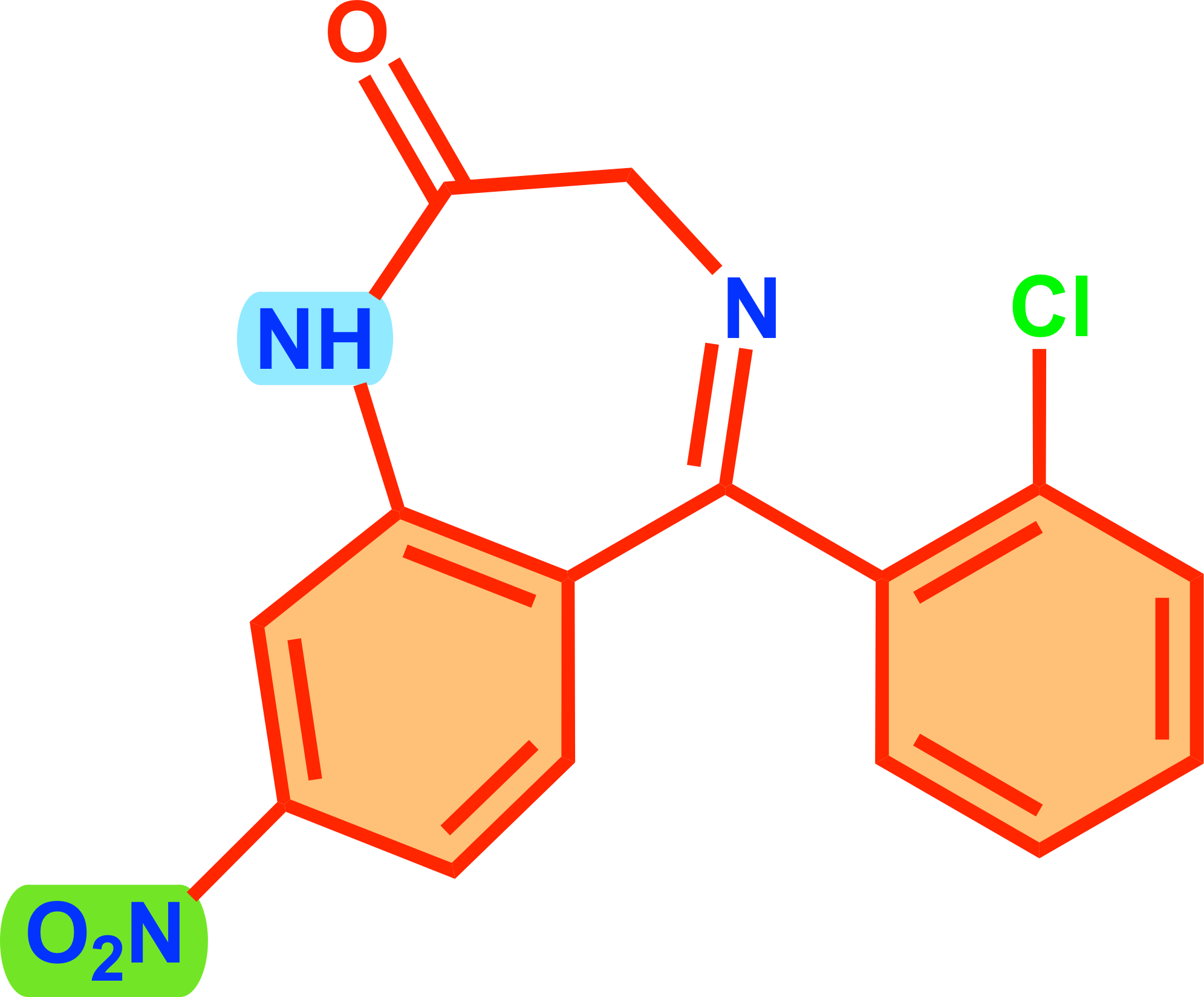

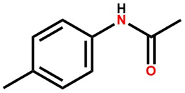

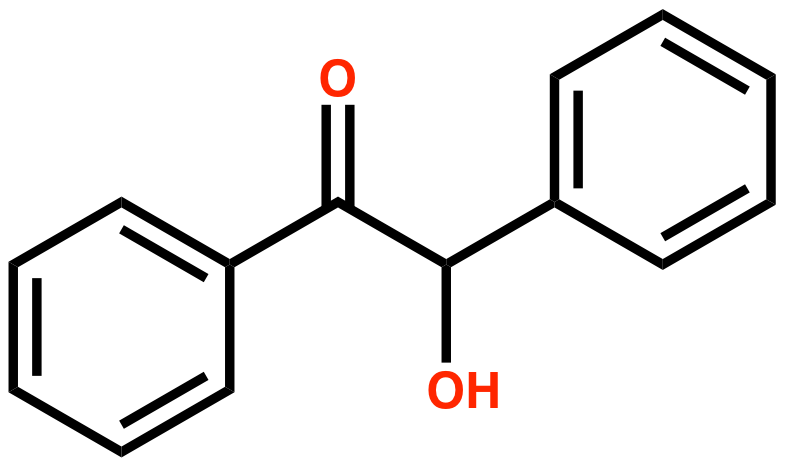
/(MPa) (×10^6^)


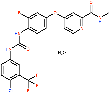


| *Clonazepam* | 28 308-338 12-30 | 3.9-72.6 | [[1](#_bookmark138)] |
| --- | --- | --- | --- |
| *4-Methyl-N-phenylacetanilide* | 21 308-328 12.16-22.5 | 38.4-480.2 | [[2](#_bookmark139)] |
| *4-Methylbenzoic acid 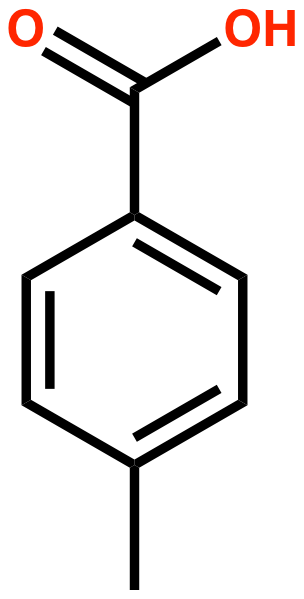* | 18 313-333 11-24.6 | 43-702 | [[3](#_bookmark140)] |
| *Benzoin* | 19 308-328 11.13-24.43 | 41-410 | [[4](#_bookmark141)] |
| 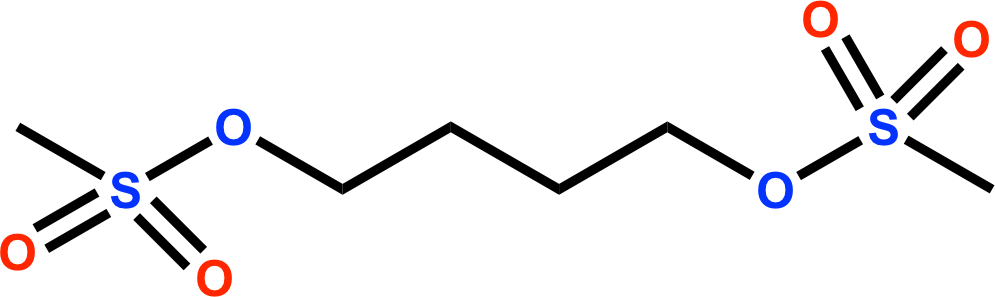*Busulfan* | 32 308-328 12-40 | 32.7-865 | [[5](#_bookmark142)] |
| 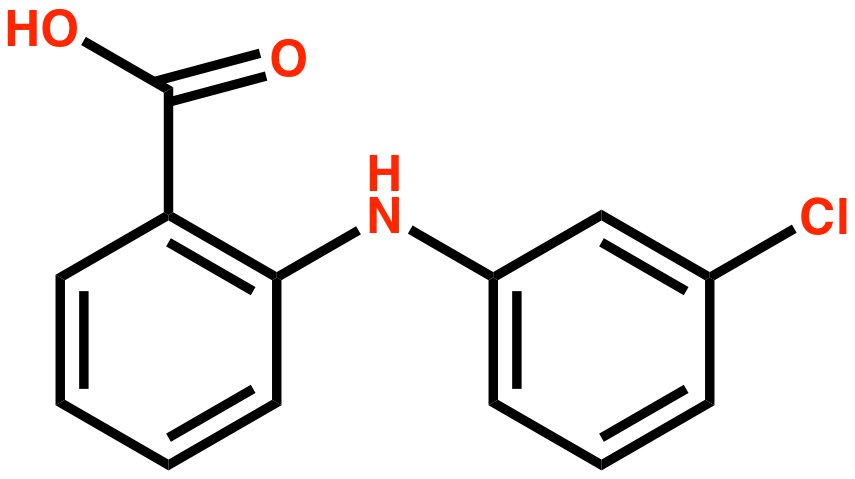*Clofenamic acid* | 24 313-333 12-36 | 1.54-35.44 | [[9](#_bookmark38)] |
| *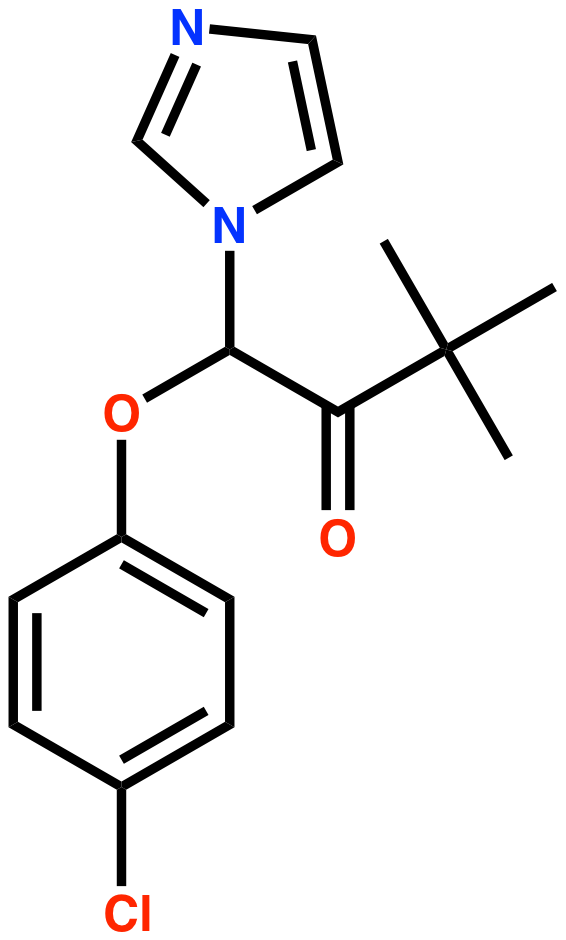Climbazole* | 24 313-333 10.55-39.89 | 6200-4880 | [[6](#_bookmark143)] |
| 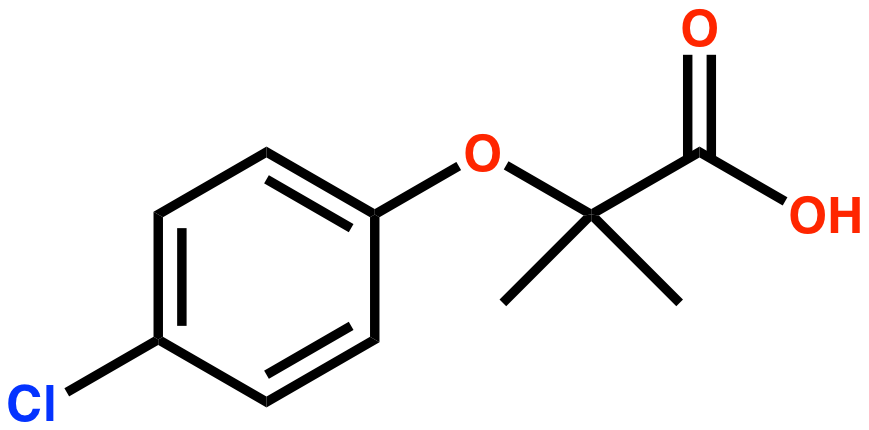*Clofibric acid* | 21 308-328 10.01-22.02 | 32.1-856 | [[7](#_bookmark144)] |
| 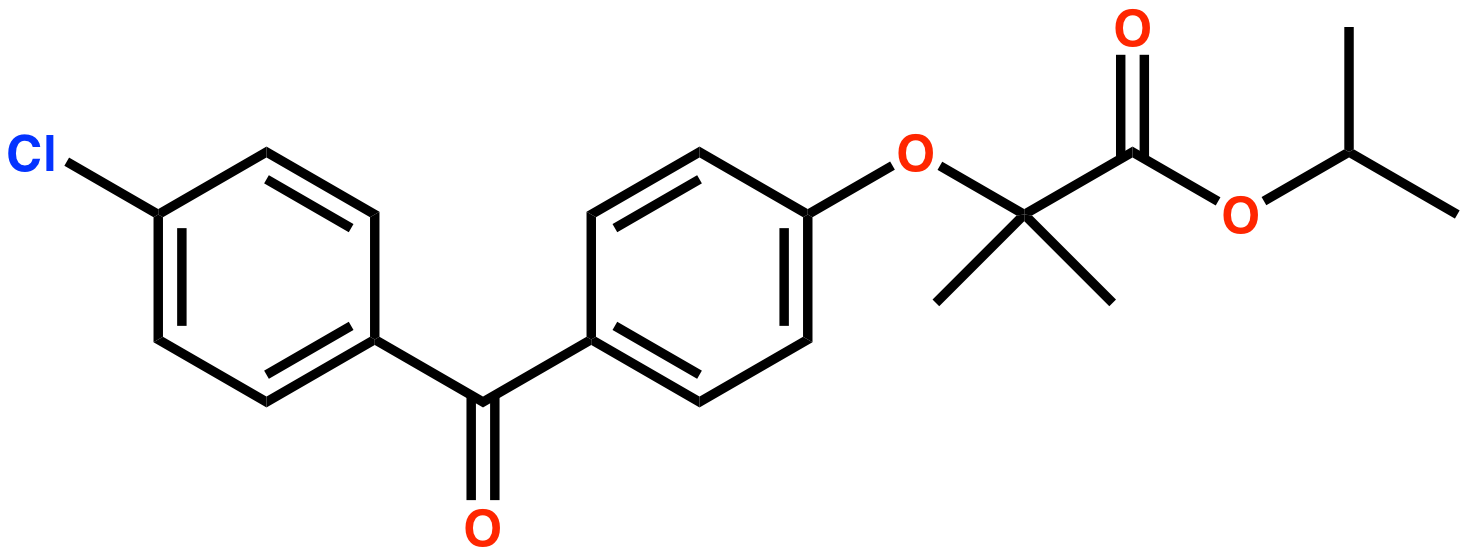*Fenofibrate* | 21 308-328 10.01-22.02 | 87.6-6660 | [[7](#_bookmark144)] |
| 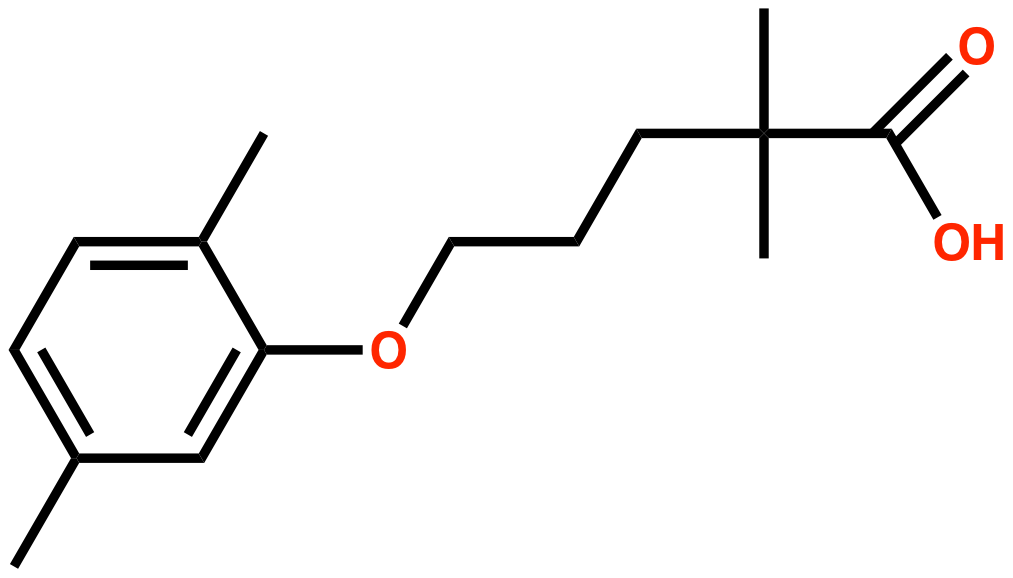*Gemfibrozil* | 21 308-328 10.01-22.02 | 29.4-4190 | [[7](#_bookmark144)] |
| 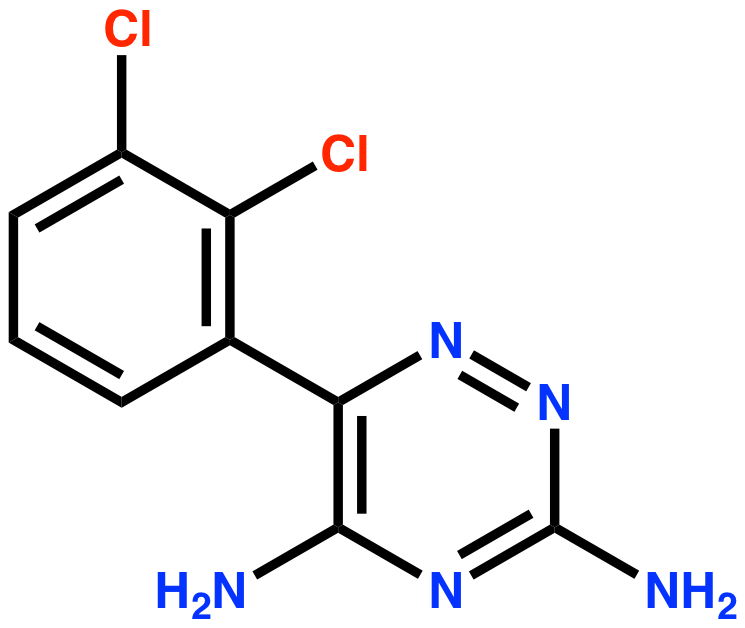  *Lamotrigine* | 36 318-338 12.16-35.46 | 0.3-5.9 | [[8](#_bookmark145)] |
| 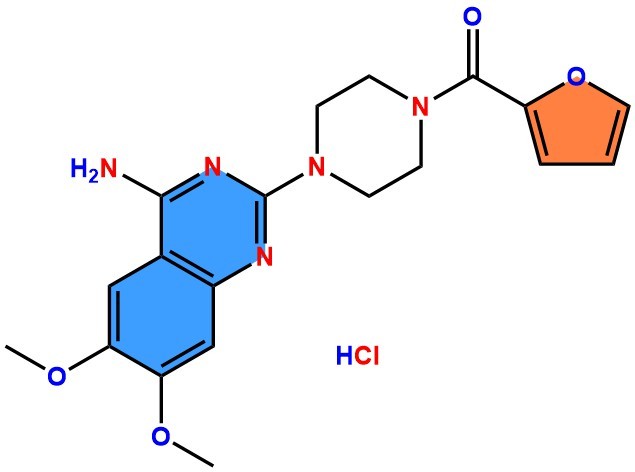  *Prazosin Hydrochloride* | 24 328-338 12-27 | 1.5-72 | [[12](#_bookmark83)] |
| 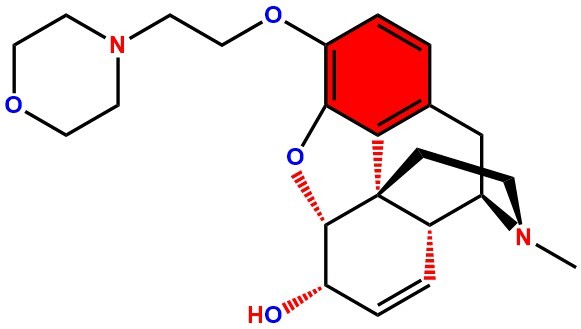*Pholcodine* | 24 328-338 12-27 | 206-593 | [[10](#_bookmark89)] |
| 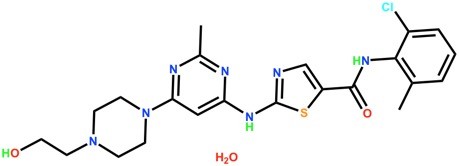*Dasatinib monohydrate* | 24 328-338 12-27 | 0.45 - 9.08 | [[11](#_bookmark90)] |
| *Regorafenib monohydrate* | 24 328-338 12-27 | 3842 -9297 | This work |

Table S2: Correlation parameters and statistical analysis ( *SSE*, *R*^2^, *R_adj_* , *RMSE*, and *AARD*%) of Rajasekhar-Madras model [[95]](#_bookmark130) Eq. ([19](#_bookmark20)) for each compound considered

| Compound | *κ* | *A*_1_ | *A*_2_ | *A*_3_ | *SSE*  (×10^10^) | *R*^2^ | *R_adj_* | *RMSE*  (×10^6^) | *AARD %* |
| --- | --- | --- | --- | --- | --- | --- | --- | --- | --- |
| *Clonazepam* | 0.901 | -4435 | 309.4 | -1.562 | 0.873 | 0.990 | 0.989 | 1.833 | 4.45 |
| *4-Methyl-N-phenylacetanilide* | 3.027 | -2646 | 50.45 | -11.83 | 174.9 | 0.853 | 0.845 | 30.34 | 17.6 |
| *4-Methylbenzoic acid* | 1.050 | -377.2 | -1.383 | -7.832 | 0.002 | 1.000 | 1.000 | 0.120 | 0.05 |
| *Benzoin* | 0.652 | -5925 | 333.6 | 6.093 | 19.30 | 0.989 | 0.988 | 10.70 | 5.49 |
| *Busulfan* | 0.861 | -6014 | 332.1 | 4.838 | 145 | 0.986 | 0.985 | 22.0 | 7.30 |
| *Clofenamic acid* | 0.124 | -7900 | 417.9 | 10.38 | 0.181 | 0.990 | 0.989 | 0.906 | 4.47 |
| *Climbazole* | 0.031 | -5430 | 494.1 | 7.089 | 7215 | 0.987 | 0.986 | 181.0 | 3.79 |
| *Clofibric acid* | -0.052 | -6955 | 402.5 | 12.70 | 93.10 | 0.991 | 0.990 | 22.10 | 3.45 |
| *Fenofibrate* | -0.203 | -7183 | 515.8 | 14.25 | 7750 | 0.990 | 0.990 | 202.0 | 4.07 |
| *Gemfibrozil* | -0.026 | -8222 | 558.8 | 15.19 | 1810 | 0.994 | 0.993 | 97.60 | 3.66 |
| *Lamotrigine* | 0.041 | -5885 | 353.2 | 3.994 | 0.005 | 0.993 | 0.992 | 0.125 | 3.64 |
| *Prazosin hydrochloride* | 1.960 | -1984 | -5.341 | -8.949 | 1.320 | 0.970 | 0.968 | 2.450 | 4.99 |
| *Pholcodine* | 3.127 | -3383 | 23.57 | -13.72 | 0.139 | 0.918 | 0.915 | 0.795 | 17.2 |
| *Dasatinib monohydrate* | 1.353 | -1868 | 91.57 | -5.458 | 217 | 0.928 | 0.925 | 31.40 | 6.86 |
| *Regorafenib monohydrate* | 1.523 | -3679 | 223.9 | -8.257 | 0.043 | 0.891 | 0.886 | 0.443 | 20.1 |
| **Mean** |  |  |  |  |  |  |  |  | **7.14** |

# References

1. R. Surya, A. Rojas, N. Esfandiari, S. Ali, Experimental study and thermodynamic modeling of clonazepam solubility in supercritical car- bon dioxide, Fluid Phase Equilibria 574 (April) (2023) 113880. [doi:](https://doi.org/10.1016/j.fluid.2023.113880) [10.1016/j.fluid.2023.113880](https://doi.org/10.1016/j.fluid.2023.113880).
2. S. Y. Huang, M. Tang, S. L. Ho, Y. P. Chen, Solubilities of N-phenylacetamide, 2-methyl-N-phenylacetamide and 4-methyl-N- phenylacetamide in supercritical carbon dioxide, Journal of Supercritical Fluids 42 (2) (2007) 165–171. [doi:10.1016/j.supflu.2007.04.001](https://doi.org/10.1016/j.supflu.2007.04.001).
3. K.-L. Tsai, F.-N. Tsai, Solubilities of Methylbenzoic Acid Isomers in Supercritical Carbon Dioxide, Journal of Chemical & Engineering Data 40 (1) (1995) 264–266. [doi:10.1021/je00017a057](https://doi.org/10.1021/je00017a057).
4. K. W. Cheng, M. Tang, Y. P. Chen, Solubilities of benzoin, propyl 4- hydroxybenzoate and mandelic acid in supercritical carbon dioxide, Fluid Phase Equilibria 201 (1) (2002) 79–96. [doi:10.1016/S0378-3812(02)](https://doi.org/10.1016/S0378-3812(02)00070-5) [00070-5](https://doi.org/10.1016/S0378-3812(02)00070-5).
5. M. Pishnamazi, S. Zabihi, S. Jamshidian, H. Z. Hezaveh, A. Z. Hezave,

S. Shirazian, Measuring solubility of a chemotherapy-anti cancer drug (busulfan) in supercritical carbon dioxide, Journal of Molecular Liquids 317 (2020) 11395.[doi:10.1016/j.molliq.2020.113954](https://doi.org/10.1016/j.molliq.2020.113954).

1. C. I. Park, M. S. Shin, H. Kim, Solubility of climbazole and triclocarban in supercritical carbon dioxide: Measurement and correlation, Journal

Chemical Thermodynamics 41 (1) (2009) 30–34. [doi:10.1016/j.jct.](https://doi.org/10.1016/j.jct.2008.08.009)

[2008.08.009](https://doi.org/10.1016/j.jct.2008.08.009).

1. Y. M. Chen, P. C. Lin, M. Tang, Y. P. Chen, Solid solubility of antilipemic agents and micronization of gemfibrozil in supercritical carbon dioxide, Journal of Supercritical Fluids 52 (2) (2010) 175–182. [doi:10.1016/j.](https://doi.org/10.1016/j.supflu.2009.12.012) [supflu.2009.12.012](https://doi.org/10.1016/j.supflu.2009.12.012).
2. M. H. Hosseini, N. Alizadeh, A. R. Khanchi, Solubility analysis of cloza- pine and lamotrigine in supercritical carbon dioxide using static sys- tem, The Journal of Supercritical Fluids 52 (1) (2010) 30–35. [doi:](https://doi.org/10.1016/j.supflu.2009.11.006) [10.1016/j.supflu.2009.11.06](https://doi.org/10.1016/j.supflu.2009.11.006).
3. H. Li, D. Jia, Q. Zhu, B. Shen, Determination, correlation and prediction of the solubilities of niflumic acid, clofenamic acid and tolfenamic acid in supercritical CO2, Fluid Phase Equilibria 392 (2015) 95–103. [doi:](https://doi.org/10.1016/j.fluid.2015.02.007) [10.1016/j.fluid.2015.02.007](https://doi.org/10.1016/j.fluid.2015.02.007).
4. G. Sodeifian, R. S. Alwi, F. Razmimanesh, Solubility of Pholcodine (an- titussive drug) in supercritical carbon dioxide: Experimental data and thermodynamic modeling, Fluid Phase Equilibria 556 (2022) 113396. [doi:10.1016/j.fluid.2022.113396](https://doi.org/10.1016/j.fluid.2022.113396).
5. G. Sodeifian, R. Surya Alwi, F. Razmimanesh, M. Abadian, Solubility of Dasatinib monohydrate (anticancer drug) in supercritical CO2: Ex- perimental and thermodynamic modeling, Journal of Molecular Liquids 346 (2022) 117899. doi:https://doi.org/10.1016/j.molliq.2021. 117899.
6. G. Sodeifian, R. Surya Alwi, F. Razmimanesh, F. Sodeifian, Solubility of prazosin hydrochloride (alpha blocker antihypertensive drug) in su- percritical CO2: Experimental and thermodynamic modelling, Journal of Molecular Liquids (2022) 119689 doi:https://doi.org/10.1016/j. molliq.2022.119689.
